# Performance Assessment of Drought Tolerant Maize Hybrids under Combined Drought and Heat Stress

**DOI:** 10.3390/agronomy8120274

**Published:** 2018-11-22

**Authors:** Silvestro Meseka, Abebe Menkir, Bunmi Bossey, Wende Mengesha

**Keywords:** combined drought and heat stress, drought tolerant, grain yield, three-way cross hybrids

## Abstract

Drought and high temperature are two major factors limiting maize productivity in sub-Saharan Africa. An increase in temperature above 30 ^°^C reduces yield by 1% under optimal rain-fed condition and by 1.7% under drought stress (DS) and up to 40% under combined drought and heat stress (DSHTS). Approaches that improve performance under the two stresses are essential to sustain productivity. The objectives of this study were to (i) assess the extent of variation in tolerance to DSHTS from among the existing best drought tolerant (DT) hybrids; (ii) examine the response patterns of the hybrids to DSHTS; (iii) identify traits that contributed to better performance under DSHTS; and (iv) select the best hybrids with tolerance to DSHTS stress. We evaluated 40 DT hybrids under DSHTS, DS, and well-watered (WW) conditions for three years. Highly significant (*p* < 0.001) differences were found among hybrids for grain yield and other traits. Moderately to low repeatability values were detected for grain yield under DS (0.63) and under DSHTS (0.48). Grain yield under DS was not correlated with grain yield under DSHTS (*r* = 0.29; *p* = 0.06), but it was correlated with grain yield under WW (*r* = 0.74; *p* < 0.001). Grain yield was strongly correlated with ears per plant, ear and pant aspects, days to anthesis and silking under both DS and DSHTS. Tassel blast accounted for 28% of the yield reduction under DSHTS. The top five DT hybrids produced 9 to 26% more grain yields than the best commercial hybrid. Three hybrids produced high grain yields under DTHTS and DS as well as under WW. These hybrids will be tested further in collaboration with partners for possible release.

## 1. Introduction

Maize (*Zea mays* L.) is a major crop for both human consumption and animal feed in Sub-Saharan Africa (SSA). Drought (DS) and heat stress (HTS) are two major limiting factors affecting maize productivity in the tropical lowlands, where erratic rains and increased temperatures are becoming of frequent occurrence [1]. It is likely that high temperatures will occur more often and will last longer and extreme precipitations events will become more intense and frequent in many regions [2]. High temperatures and changes in rainfall pattern can cause significant decline in maize yields under rainfed conditions in the tropical region with SSA being one of the worst affected areas [3,4]. An increase in temperature over 30 ^°^C reduces grain yield by 1% under optimal rain-fed condition and by 1.7% under DS [5] and up to 40% under combined drought and heat stress [6].

With the current changes in climatic conditions, it is projected that by 2030 drought and higher temperatures may render 40% of the current maize growing areas in Africa unsuitable for varieties available today [7]. Unless strong adaptation measures are taken, these changes are expected to reduce yields of maize and other food crops by 10% to 20%, causing marked decline in human welfare [8,9]. Adaptation to climate change may involve the use of crop varieties that are endowed with tolerance to higher temperatures and drought, and resistance to emerging pests and diseases [9]. Approaches that improve the performance of maize genotypes under combined drought and heat stress (DSHTS) are therefore essential to sustain productivity and to avoid widespread famine in Africa.

Maize is more sensitive than other cereals to drought at flowering because anthers and silks are separated by about 1 m, and pollen and stigma are exposed to the environment [10,11]. The crop is particularly sensitive to DS one week before and two weeks after flowering [12,13], resulting in an average yield loss of 20% to 50% [14]. On the other hand, HTS in maize is associated with temperatures rising above an optimum of 30 ^°^C that adversely affect physiological processes [15], resulting in increased respiration, reduced life cycle, light interception, photosynthesis, and increased pollen sterility [16]. High temperature two weeks before flowering causes permanent tissue injury to developing leaves resulting in leaf firing [15], accelerate the rate of leaf senescence [17], and premature lodging. High temperature occurring at the on-set of flowering causes tassel blasting that reduces pollen production, pollen viability and pollination rate [18,19]. At grain-filling stage, high temperatures lead to slow and short grain-filling period, reduced kernel set, and grain weight [18–20], resulting in 10% reduction in grain yield [21].

Drought and heat stress occur simultaneously in farmers’ fields when the growing season is hot, and the distribution of rains is erratic. Heyne & Brunson [22] showed that the combined effect of drought and heat stress in maize was much greater than the effect of each stress separately. The simultaneous occurrence of drought and heat stresses in farmers’ fields are becoming increasing common in the tropical lowlands, particularly in maize growing areas in West Africa and Sahelian regions [1] and Eastern and Southern Africa [4]. These regions are known to be vulnerable to climate changes due to high climate variability, high reliance on rainfed agriculture, and limited economic and institutional capacity to respond to climate change [1]. Depending on the timing, duration and severity, yield losses in maize due to DSHTS can vary from 15% to 40% and they can exceed 70% [6,23]. Heiniger [24] reported that DSTHTS occurring during pollination can cause up to 100% yield loss in maize. Tassel is more susceptible to heat than drought stress because of its position that exposes pollen to heat, whereas drought is more detrimental to silk than heat stress causing delay in silk emergency [3,25]. Sullivan & Blum [26] observed that maize closes its stomata sooner than sorghum (*Sorghum bicolor* L.) under DS thereby increasing the canopy temperature by more than 9 ^°^C [27]. Maize genotypes capable of keeping their stomata partially open may maintain enough transpirational cooling to avoid excessively high leaf temperature.

The breeding strategies in maize that have been adopted so far have focused on improvement of each stress separately. Preliminary results of hybrids evaluated under the Drought Tolerant Maize for Africa (DTMA) project revealed that tolerance to DS does not necessarily confer tolerance to HTS or DSHTS, which have implications to breeding for adaptation to climate change in maize production systems in SSA [4]. Understanding the response of hybrids selected for drought tolerance (DT) to DSHTS may thus facilitate the identification of promising hybrids with tolerance to DSHTS fairly quickly for further testing, release and deployment. Parents of these hybrids can also be screened under DSHTS to identify source materials for breeding to further increase their tolerance to much higher levels. This study was, therefore, conducted to (i) assess the extent of variation in tolerance to DSHTS from among the existing best DT hybrids; (ii) examine the response patterns of the hybrids to DSHTS; (iii) identify traits that contributed to better performance under DSHTS; and (iv) select the best hybrids with tolerance to DSHTS stress.

## 2. Materials and Methods

### 2.1 Genetic Material and Experimental Design

The genetic material that was used in this study consisted of 29 promising DT three-way cross maize hybrids, 10 commercial hybrid checks, and a local variety commonly grown by farmers in the area. These hybrids have been evaluated in regional trials in partnership with the National Agricultural Research System (NARS) in West and Central Africa (WCA) for over three years (Table 1).

**Table 1:** List of drought tolerant maize germplasm including a local check variety used for assessing performance under combined drought and heat stress at Kadawa in Nigeria. M: designation of regional maize trial; CH: commercial hybrid.

Entry	Name	Description	Entry	Name	Description
1	M0826-1	DT hybrid	21	M1227-9	DT hybrid
2	M0826-2	DT hybrid	22	M1227-10	DT hybrid
3	M0826-3	DT hybrid	23	M1227-11	DT hybrid
4	M0826-4	DT hybrid	24	M1227-12	DT hybrid
5	M1124-18	DT hybrid	25	M1227-15	DT hybrid
6	M1124-15	DT hybrid	26	M1227-17	DT hybrid
7	M1124-16	DT hybrid	27	M1227-18	DT hybrid
8	M1124-17	DT hybrid	28	M1227-19	DT hybrid
9	M1124-23	DT hybrid	29	M1227-20	DT hybrid
10	M1124-24	DT hybrid	30	CH1	Commercial hybrid
11	M1124-29	DT hybrid	31	CH2	Commercial hybrid
12	M1124-26	DT hybrid	32	CH3	Commercial hybrid
13	M1124-27	DT hybrid	33	CH4	Commercial hybrid
14	M1124-31	DT hybrid	34	CH5	Commercial hybrid
15	M1124-28	DT hybrid	35	CH6	Commercial hybrid
16	M1227-3	DT hybrid	36	CH7	Commercial hybrid
17	M1227-5	DT hybrid	37	CH8	Commercial hybrid
18	M1227-6	DT hybrid	38	CH9	Commercial hybrid
19	M1227-7	DT hybrid	39	CH10	Commercial hybrid
20	M1227-8	DT hybrid	40	Local check	Local variety

### 2.2 Weather Conditions during the Trial

**Figure 1:**
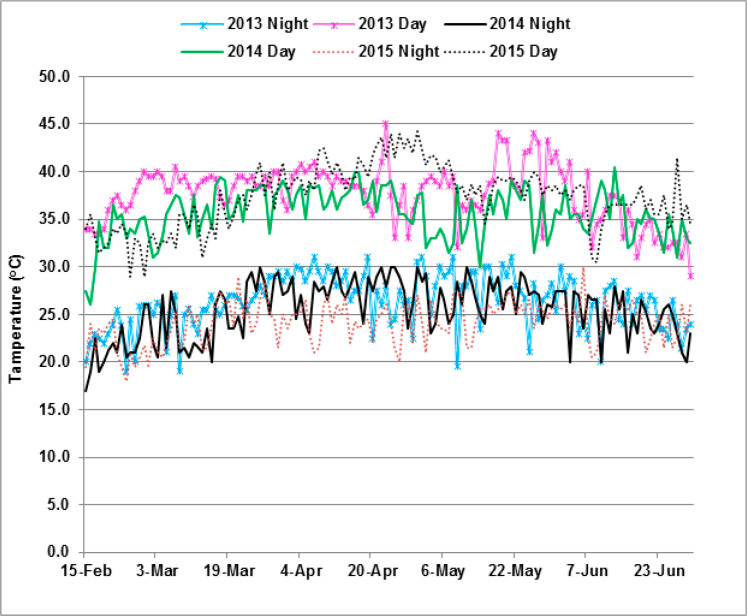
Average day and night temperatures recorded during the growing period at Kadawa.

The trial under DSHTS was conducted during the dry season in 2013, 2014, and 2015. In each year, the lowest day and night temperatures were recorded in February at the time of planting and the highest in April. During evaluation of this trial, there were incidences of rains in the months of April and May, but the amounts were not sufficient to have significant effects on the intensity of drought stress under elevated temperature. In each year, the peak of high temperature occurred in April (Figure 1), which coincided with the reproductive and grain-filling stages of the maize crop. During this period, the maximum day and night temperatures were 45 and 31 ^°^C, respectively, which were detrimental to fertilization, seed setting, and grain-filling in maize. Minimum day and night temperatures of ≤27 ^°^C and ≤20 ^°^C, respectively, are considered to be optimal for the growth and development of maize in the tropical lowlands.

### 2.3 Experimental Design

The 40 hybrids including a local check was arranged in 8 × 5 alpha lattice design (incomplete design) with three replications evaluated for three years (2013–2015) under DSHTS and for two years (2014 and 2015) under DS and WW. Each replicate consisted of eight incomplete blocks with five plots in each block. In each year, the genetic materials, experimental design, and replications were the same. Each entry was planted in a two-row 5 m plot spaced 0.75 m apart with 0.50 m spacing between hills within a row. Three seeds were planted in a hill and thinned to two plants per hill three weeks after sowing to attain a population density of 53,000 plants per hectare (ha^−1^). A compound fertilizer was applied at the rates of 60 kg N, 60 kg P, and 60 kg K ha^−1^ at the time of planting. An additional 60 kg N ha^−1^ was applied as top dressing four weeks later. Gramoxone and atraxine were applied as pre-immergence herbicides at the rates of 1.5 L gramoxone and 2.5 L atraxine in 200 L of water ha^−1^. Subsequent manual weeding was done to keep the trials weed free.

### 2.4 Evaluation under Combined Drought and Heat Stress

The 40 maize hybrids were evaluated for tolerance to DSHTS at Kadawa (11^°^39^0^ N, 8^°^27^0^ E, elevation 500 m) in Kano State in Nigeria, where hot and dry weather occur between February and June. The soil type is Regosols, with mainly sandy to clay loam texture, pH of 5.9, organic carbon 4.3 g kg^−1^, and residual nitrogen 0.24 g kg^−1^ [28]. In Kadawa, the temperature during the dry season ranges from 33 to 45 ^°^C. This helped in conducting this trial under DSHTS, whereby water supply was carefully controlled through furrow irrigation. The flowering and grain filling periods were generally in April when rainfall incidences were negligible and relative humidity ranged from 15% to 46%, allowing for exposure of the hybrids to drought and elevated temperatures (Figure 1).

Maize seeds were planted in mid-February for three consecutive years (2013 to 2015) and flowered in mid-April, the hottest period at Kadawa. The trial was initially grown under well-watered conditions prior to exposure to drought and heat stress. A gravity irrigation system was used to supply sufficient water through furrows to the crop every four days during the first 45 days after planting. Thereafter, irrigation was withdrawn in mid-April when mean day temperature ranged from 36 to 45 ^°^C and night temperature varied from 27 to 30 ^°^C, inducing the combined effects of drought and heat stress that coincided with flowering. Irrigation was resumed after 21 days and it was applied only once a week to allow grain filling of the crop until physiological maturity. Meteorological data were recorded during the trial period using an automated weather station at Bagauda in Kano State. As shown in Table 2, the amount of precipitation received during the period when the trial was exposed to heat and drought stress each year was very small.

**Table 2:** Average monthly temperature and rainfall recorded from a weather station at Bagauda in Nigeria during the growing period in 2013, 2014, and 2015.

Month	2013			2014			2015		
	Night (*^°^*C)	Day (*^°^*C)	Rainfall (mm)	Night (*^°^*C)	Day (*^°^*C)	Rainfall (mm)	Night (*^°^*C)	Day (*^°^*C)	Rainfall (mm)
February	21	34	0	20	33	0	22	33	0
March	26	39	0	24	37	0	24	36	2
April	27	43	6	27	40	21	24	41	0
May	28	39	33	25	38	17	25	39	60
June	26	35	189	24	36	156	24	36	88

### 2.5 Evaluation under Drought Stress

The same set of hybrids were evaluated under controlled DS and well-watered (WW) conditions at Ikenne (6^°^52^0^ N, 3^°^43^0^ E, altitude 60 m) in Nigeria during the dry season. The experiment was conducted in two adjacent blocks separated by 50 m to prevent spill-over of irrigation water. Maize seeds were planted in mid-November in 2014 and 2015, so that flowering occurs in mid-January where incidences of rainfall were minimal. A sprinkler irrigation system was used to provide sufficient water every week during the first 35 days after planting. Thereafter, irrigation was withdrawn from DS block to induce drought stress at flowering and grain filling stages, allowing for the crop to mature on stored soil moisture. The WW block continued to receive irrigation once a week until physiological maturity.

### 2.6. Data Collection

Data were recorded on different traits in each plot. Days to anthesis and silking were recorded as the number of days from planting to when 50% of the plants in a plot were shedding pollen and had emerged silks, respectively. Data on tassel blast (TB) and barren plants (BP) were only recorded on DSHTS trial. TB was rated during flowering on a scale of 1 to 5, where 1 = few plants showing tassel blast and 5 = all plants showing severe tassel blast, whereas BP was rated on a scale of 1 to 5, where 1 = few plants without ears and 5 = more than 50% or all plants bearing no ears recorded in each plot two weeks after flowering. Anthesis to silkin interval (ASI) was computed as a difference between days to silking and anthesis. Plant and ear heights were recorded on five randomly selected plants, as distance from the ground to the base of the tassel and the upper ear, respectively. Leaf death score (leaf senescence) was rated on a scale of 1 to 10, where 1 = 10% dead leaf area and 10 = 100% dead leaf area recorded only in DS and DSHTS trials. Number of ears per plant (EPP) was computed as the proportion of the total number of ears harvested divided by the total number of plants at harvest. Plant aspect was visually scored on a scale of 1 to 5, where 1 = excellent overall phenotypic appeal and 5 = poor overall phenotypic appeal. Similarly, ear aspect was scored on a scale of 1 to 5, where 1 = clean, uniform, large, and well-filled ears and 5 = rotten, variable, small, and partially filled ears. All ears harvested from each plot were shelled and used to determine the percentage grain moisture and grain weight. Grain yield adjusted to 15% moisture was computed from the grain weight recorded form each plot.

### 2.7. Data Analysis

The analysis of data was performed using a mixed model procedure in SAS [29] version 9.4, following the standard linear mixed model that was described by Vargas et al. [30]. All effects except genotypes were considered random and Best Linear Unbiased Predictor (BLUP) was computed for all traits using the procedure of Robinson [31]. Repeatability estimates were computed to determine the precision of measuring traits in the trials.

Cluster analysis using Euclidean distance with standardized values of agronomic data was computed to classify the DT hybrids into groups. A dendrogram was constructed using the Unweighted Pair Group Method with Arithmetic means (UPGMA) and the resulting tree was used to determine the association between the DT hybrids under DS and DSHTS. Phenotypic coefficients of correlations for grain yield with secondary traits were computed to further determine the secondary traits contributing to grain yield. Grain yields and TB scores were plotted on a graph to show the trend of yields of the hybrids under DSHTS conditions.

## 3. Results

### 3.1. Performance of Hybrids

In the combined analysis of variance across years (Table 3), year had significant effect on all the traits recorded on hybrids. Significant differences (*p* < 0.001) were found among hybrids for all of the traits measured under DSHTS. Hybrid x year interaction was significant for grain yield and seven of the other 10 traits. Repeatability values for the measured traits under DSHTS ranged from 0.13 for TB to 0.73 for plant height. Mean grain yields of the hybrids varied from 1016 kg ha^−1^ for a commercial hybrid CH2 to 3453 kg ha^−1^ for DT hybrid M1227-17. The top five DT hybrids produced 9% to 26% more grain yields than the best commercial hybrid check CH9 and out-yielded the local check by 56% to 64%. Most of the hybrids were significantly (*p* < 0.05) better than the local check in plant and ear aspect scores, had more EPP and fewer TB and BP.

**Table 3:** Mean sum of squares from combined analysis of variance of 40 drought tolerant maize hybrids evaluated under combined drought and heat stress in 2013, 2014, and 2015 at Kadawa in Nigeria.

Source	DF	Grain Yield (kg/ha)	Silking (day)	Plant Height (cm)	Ear Height (cm)	Ear per Plant (No.)	Ear Aspect (1–5) ^a^	Plant Aspect (1–5) ^b^	Leaf Death1 (1–10) ^c1^	Leaf Death2 (1–10) ^c2^	Tassel Blast (1–5) ^d^	Barren Plant (1–5) ^e^
Year	2	95,869,279 ^***^	4009 ^***^	26727 ^***^	6199 ^***^	12.53 ^***^	12.58 ^***^	12.57 ^***^	46.88 ^***^	71.68 ^***^	2.64 ^***^	9.06 ^***^
Block (Rep^*^Year)	69	4,517,557 ^***^	35 ^***^	1121 ^***^	537 ^***^	0.13 ^***^	1.10 ^***^	0.34 ^***^	0.62 ^***^	1.88 ^***^	0.29 ^***^	0.28 ^***^
Genotype	39	2,709,746.6 ^***^	26 ^***^	1118 ^***^	571 ^***^	0.14 ^***^	0.74 ^***^	0.55 ^***^	0.88 ^***^	1.64 ^**^	0.45 ^***^	0.22 ^**^
Genotype x Year	77	119,982,477 ^**^	11 ^**^	306	169 ^*^	0.06 ^**^	0.39 ^*^	0.33 ^***^	0.23	1.23 ^**^	0.37 ^***^	0.15
Residual	166	820,563	8	240	113	0.03	0.25	0.17	0.22	0.73	0.14	0.11
Repeatability		0.48	0.49	0.73	0.71	0.64	0.52	0.48	0.63	0.38	0.13	0.40

*, **, *** Significant at 0.5, 0.01 and 0.001 probability levels, respectively; DF = degree of freedom;

a Ear aspect, scored on a scale of 1–5, where 1 = uniform ear filled and grain color and 5 = poor ear filling with multiple grain colors;

b Plant aspect, a visual score on a scale of 1–5, where 1 = good phenotypic appeal and 5 = poor phenotypic appeal;

c1 First leaf senescence scored on a scale of 1–10, where 1 = 10% leaf death and 10 = 100% leaf death;

c2 Second leaf senescence scored on a scale of 1–10, where 1 = 10% leaf death and 10 = 100% leaf death;

d Tassel blast scored on a scale on a scale of 1–5, where 1 = few tassel blast and 5 = dry tassel;

e Barren plants scored on a scale of 1–5, where 1 = few barren plants and 5 = all plants without ears.

Results of combined analysis across two years under DS (Table 4) revealed significant differences among the DT hybrids for grain yield and five of the eight other traits. The interaction of the hybrids with year was also significant for grain yield and four other traits. Repeatability values ranged from 0.25 for plant aspect to 0.79 for second leaf death score. Mean grain yield of the hybrids varied from 1199 kg ha^−1^ for commercial hybrid CH2 to 3151 kg ha^−1^ for DT hybrid M1124-17. The best commercial hybrids (CH4 and CH5) were competitive with the best DT hybrids under DS. Under WW conditions, differences among the DT hybrids were significant for grain yield and most traits. Hybrid x year interaction was significant for days to silking, plant height, plant, and ear aspect scores. Repeatability values ranged from 0.28 for ASI to 0.85 for days to silking. Mean grain yields of the hybrids ranged from 1272 kg ha^−1^ for local check to 5126 kg ha^−1^ for DT hybrid M1227-5.

**Table 4:** Mean sum of squares from combined analysis of variance of 40 drought tolerant maize hybrids evaluated in 2014 and 2015 under drought stress and well-watered conditions at Ikenne in Nigeria.

Source	DF	Grain Yield (kg/ha)	Days to Silking (day)	ASI ^†^ (day)	Plant Height (cm)	Ear Height (cm)	Ear per Plant (No.)	Ear Aspect (1–5) ^a^	Plant Aspect (1–5) ^b^	Leaf Death2 (1–10) ^c2^
Drought stress										
Year	1	17,474,594 ^***^	127.7 ^***^	3.60 ^**^	13250 ^***^	7576 ^***^	3.43 ^***^	9.00 ^*^	92.64 ^***^	0.25
Block (Rep^*^Year)	30	304,216	2.5 ^*^	0.71	405 ^*^	190 ^*^	0.14 ^*^	2.20	1.94 ^**^	3.79
Genotype	39	1,133,984 ^***^	8.6 ^***^	0.51	271	112	0.24 ^***^	8.00 ^***^	5.19 ^***^	21.90 ^***^
Genotype x Year	39	467,734 ^**^	2.2 ^*^	0.40	177	90	0.12^*^	5.61 ^**^	3.71 ^***^	5.38
Residual	50	218,768	1.2	0.50	202	106	0.07	1.98	0.71	4.33
Repeatability		0.63	0.77	0.17	0.28	0.0	0.59	0.31	0.25	0.79
Well-watered										
Year	1	21,933,107 ^***^	0.4	1.00	11833 ^***^	837 ^**^	0.10	33.06 ^***^	112.89 ^***^	-
Block (Rep^*^Year)	30	373,970	1.1	2.29	205 ^**^	138 ^*^	0.12	2.44 ^*^	2.11 ^*^	-
Genotype	39	2,134,240 ^***^	9.5 ^***^	3.04	455 ^***^	227 ^***^	0.23 ^*^	6.99 ^***^	4.69 ^***^	-
Genotype x Year	30	589,706	1.6 ^**^	2.94	146 ^*^	73	0.11	3.69 ^**^	2.18 ^**^	-
Residual	50	533,513	0.8	3.02	80	67	0.12	1.39	1.06	-
Repeatability		0.76	0.85	0.28	0.72	0.68	0.59	0.61	0.60	-

*, **, *** Significant at 0.5, 0.01 and 0.001 probability levels, respectively; DF = degree of freedom;

† Anthesis to silking interval; - Trait not recorded under well-watered condition;

a Ear aspect, scored on a scale of 1–5, where 1 = uniform ear filled and grain color and 5 = poor ear filling with multiple grain colors;

b Plant aspect, a visual score on a scale of 1–5, where 1 = good phenotypic appeal and 5 = poor phenotypic appeal;

c2 Second leaf senescence scored on a scale of 1–10, where 1 = 10% leaf death and 10 = 100% leaf death.

### 3.2. Association of Traits under Optimum, Drought and Combined Drought and Heat Stress

The correlations of grain yield with other agronomic traits measured in this study are presented in Table 5. Grain yield was strongly and positively correlated with EPP and plant aspect scores under the three treatments. Significant and negative correlations were detected between grain yield and days to anthesis and silking only under DS and DSHTS. Grain yield was significantly and negatively correlated with ASI (*r* = −0.60; *p* < 0.0001) only under DS. A significant and negative relationship was detected between grain yield and BP and TB only under DSHTS.

**Table 5:** Coefficient of phenotypic correlations between grain yield and other agronomic traits measured under well-watered, drought, and combined drought and heat stress conditions.

Trait	DSHTS	DS	WW
Days to anthesis	−0.42 ^**^	−0.40 ^**^	−0.09
Days to silking	−0.40 ^**^	−0.48 ^**^	−0.13
Anthesis–silking interval	−0.17	−0.59 ^***^	−0.30 ^*^
Plant height	0.07	0.11	0.14
Ear height	−0.10	−0.03	0.13
Ears per plant	0.62 ^***^	0.68 ^***^	0.65 ^***^
Ear aspect	−0.85 ^***^	−0.83 ^***^	−0.84 ^***^
Plant aspect	−0.47 ^**^	−0.70 ^***^	−0.87 ^***^
Leaf death1	−0.20	-	-
Leaf death2	−0.02	−0.25 ^*^	-
Barren plant	−0.51 ^**^	-	-
Tassel blast	−0.42 ^**^	-	-

*, **, *** Significant at 0.5, 0.01 and 0.001 probability levels, respectively; - Trait not recorded under well-watered condition; DS, drought tolerant; DSHTS, combined drought and heat stress; WW, well-watered.

Results of association of the same traits under DSHTS and DS as well as under WW conditions are presented in Table 6. Grain yield under DS condition had strong correlation with grain yield under WW condition (*r* = 0.74; *p* < 0.001). However, the correlation between grain yield under DSHTS and that under DS condition (*r* = 0.29; *p* = 0.0689) as well as that under WW condition (*r* = 0.19; *p* = 0.2313) were weak. ASI, EPP, days to anthesis, and silking under DSHTS were significantly correlated with ASI, EPP, days to anthesis, and silkiing interval under DS and WW conditions. A similar trend was observed between EPP, ASI, days to anthesis, and silking recorded under DS with the same set of traits being measured under the WW condition. Ear and plant aspect scores under DS were strongly correlated with ear and plant aspect scores under WW.

**Table 6:** Association of traits measured under combined drought and heat stress with same traits under drought and well-watered conditions as well as those measured under drought stress with same traits under well-watered conditions.

Trait	DSHTS vs. DS	DSHTS vs. WW	DS vs. WW
Grain yield	0.29 ^*^	0.19	0.74 ^***^
Anthesis	0.58 ^***^	0.64 ^***^	0.89 ^***^
Silking	0.64 ^***^	0.65 ^***^	0.90 ^***^
Anthesis-silning	0.45 ^**^	0.45 ^**^	0.67 ^***^
Ears per plant	0.47 ^**^	0.52 ^**^	0.44 ^**^
Ear aspect	0.26 ^*^	0.06	0.67 ^***^
Plant aspect	0.19	0.23	0.54 ^**^
Leaf death1	0.18	-	-

*, **, *** Significant at 0.5, 0.01 and 0.001 probability levels, respectively; - Trait not recorded under well-watered condition; DS, drought stress; DTHTS, combined drought and heat stress; WW, well-watered.

**Figure 2:**
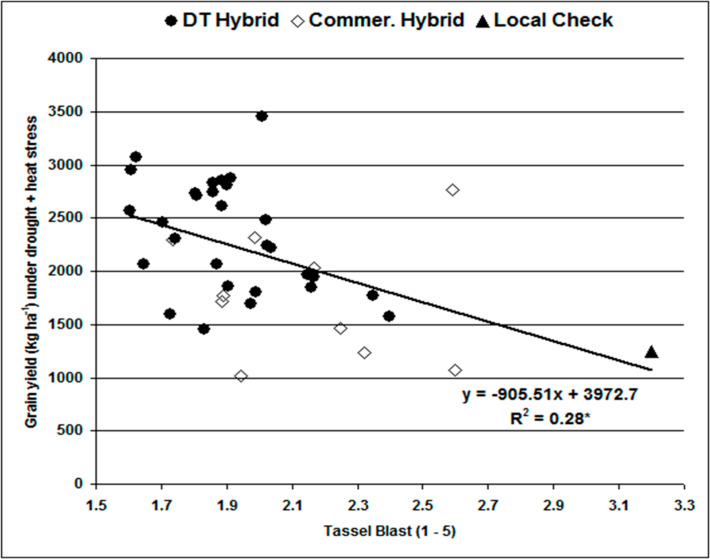
Regression of grain yield (kg ha−1) of 29 drought tolerance (DT) hybrids, nine commercial hybrids, and a local check on tassel blast under combined drought and heat stress.

Regression of grain yield on TB scores showed that hybrids with high TB scores had low grain yields (Figure 2). TB accounted for 28% of yield reduction under DSHTS. Generally, commercial hybrids and the local check had high TB and low grain yields (<2000 kg ha^−1^). Most of the DT hybrids, including two commercial hybrid checks (CH8 and CH9), had low TB and produced >2000 kg ha^−1^ grain yields under DSHTS. This trend was also observed when grain yield was regressed on mean day temperatures during the critical flowering and grain filling stages (results not shown). For every 1 ^°^C increase in temperature at flowering and grain filling period, grain yield dropped by 30 kg ha^−1^ accounting for 3.53% grain yield variation.

### 3.3. Grouping of DT Hybrids under Combined Drought and Heat Stress

**Figure 3:**
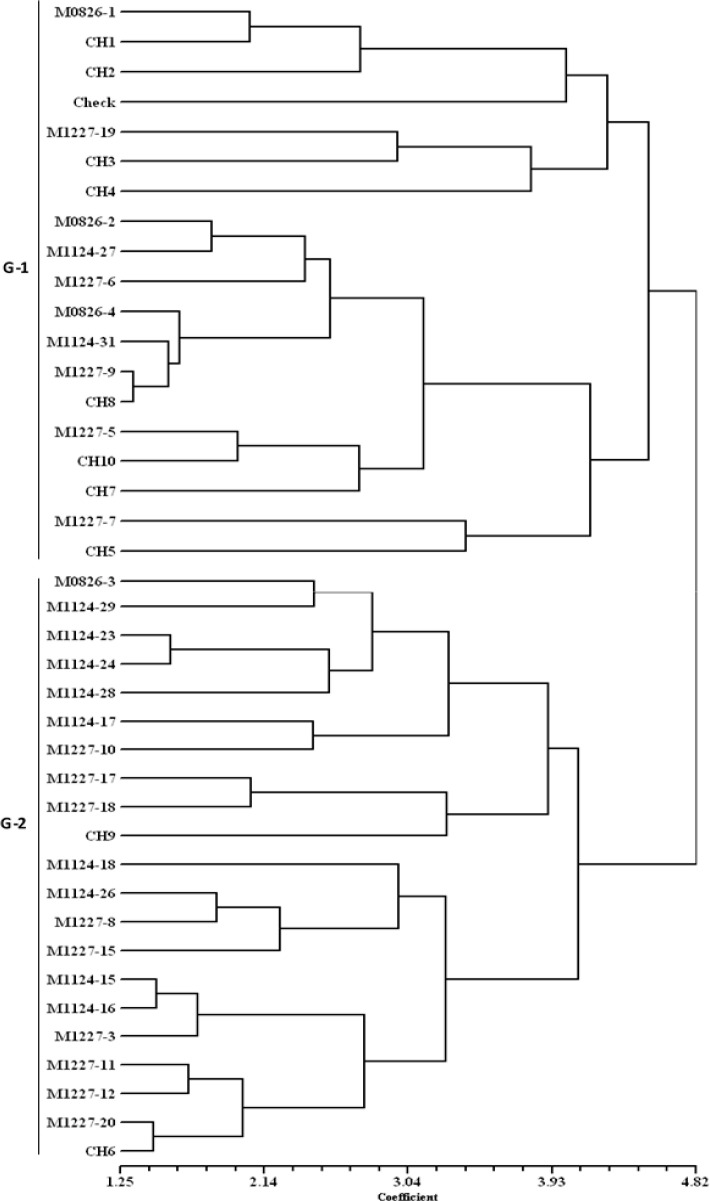
Dendrogram of 29 DT hybrids, 10 commercial hybrids, and a local check obtained using cluster analysis of Euclidean distance matrix under combined drought and heat stress.

A dendrogram generated from the standardized data of grain yield, plant and ear aspect scores, EPP and ASI is presented in Figure 3. Under DSHTS, the hybrids were classified into two major groups (G-1 and G-2). The first group consisted of 19 hybrids, including seven DT hybrid eight commercial hybrids (CH1, CH2, CH3, CH4, CH5, CH7, CH8, and CH10), three released hybrids (M0826-2, M0826-3, and M0826-4), and the local check. Most hybrids in this group had low grain yields, high number of BP and TB. The second group consisted of 21 hybrids involving 18 DT hybrids, two commercial hybrids (CH6 and CH9), and a released hybrid (M0826-3). Most hybrids in this group had high grain yields, good plant type, few BP and less TB, but poor ear aspect scores.

### 3.4. Grouping of DT Hybrids under Drought Stress

**Figure 4:**
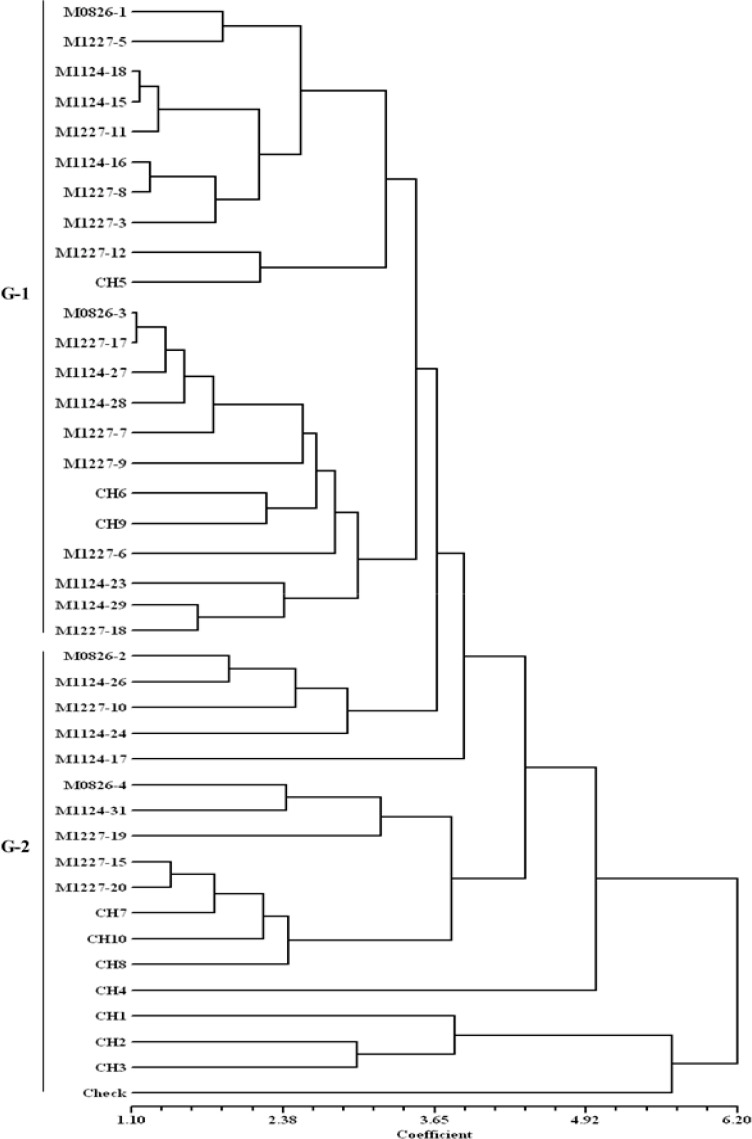
Dendrogram of 29 DT hybrids, 10 commercial hybrids, and a local check obtained using cluster analysis of Euclidean distance matrix under drought stress.

The cluster analysis classified the 40 hybrids into two major groups under DS (Figure 4). The first group contained 27 hybrids, involving 21 DT hybrids, three commercial hybrids (CH5, CH6, and CH9), and three released hybrids (M0826-2, M0826-3, and M0826-4). Most hybrids in this group combined high grain yield with short ASI and good ear aspect scores and had relatively good plant type. The second group contained 13 hybrids, including four DT hybrids, seven commercial hybrids (CH1, CH2, CH3, CH4, CH7, CH8, and CH10), a released hybrid (M0826-4), and a local check. Most hybrids in this group had low grain yields, long ASI, poor plant type, and ear aspect scores.

### 3.5. Performance of the Top 10 DT and Commercial Hybrids under Stress and Optimum Conditions

The top 10 DT hybrids selected under DSHTS produced grain yields ranging from 2607 kg ha^−1^ for M1124-29 to 3453 kg ha^−1^ for M1227-17 (Table 7). Most of the top hybrids also produced grain yields more than 2400 kg ha^−1^ under DS. The hybrids produced 11% more grain yields under DSHTS than under DS. These hybrids, on average, attained days to silking at 68 days after planting, had good ear and plant aspect scores. Three hybrids (M1124-18, M1227-15, and M1227-8) had longer stay-green with leaf death scores ranging from 6.5 to 6.8. Although the top 10 hybrids selected under DS produced grain yields ranging from 2757 kg ha^−1^ to 3151 kg ha^−1^, they produced low grain yields under DSHTS. However, two of the top 10 hybrids (M1124-24 and M1227-3) selected under DS were also found among the 10 best hybrids under DSHTS (Table 7).

**Table 7:** Mean grain yield of the ten top DT hybrids, nine commercial hybrids, and a check variety averaged for three years (2013–2015) under DSHTS and for two years (2014 and 2015) under WW and DS. Means of days to silking, ear per plant, ear aspect, plant aspect, leaf death1, leaf death2, TB, and BP averaged for three years under DSHTS.

Hybrid	Grain Yield (kg/ha)			Silking (Day)	Ear Plant (No.)	Ear Aspect (1–5) ^a^	Plant Aspect (1–5) ^b^	Leaf Death1 (1–10) ^c1^	Leaf Death2 (1–10) ^c2^	TB (1–5) ^d^	BP (1–5) ^e^
	DSHS	DS	WW								
Top 10 DT hybrids											
M1227-17	3453	2449	3483	67	0.9	2.8	3.0	4.3	8.0	1.7	1.9
M0826-3	3071	2440	4366	68	0.9	3.2	3.1	3.8	7.1	1.6	2.0
M1124-18	2951	2641	4390	70	1.2	3.2	2.8	4.1	6.6	1.6	2.0
M1124-24	2854	3052	3897	65	0.9	3.3	2.9	3.7	7.0	1.9	2.0
M1227-18	2832	2752	4207	68	0.9	2.8	2.8	3.7	8.1	1.9	1.9
M1124-23	2812	2737	4427	65	0.9	3.2	2.9	4.1	7.3	1.9	1.9
M1227-3	2745	2757	4186	68	1.1	3.3	2.6	4.3	7.6	1.9	1.8
M1227-8	2708	2601	3894	70	1.0	3.1	3.1	4.3	6.5	1.8	2.0
M1124-29	2607	2756	3280	67	0.7	2.9	2.9	4.2	7.7	1.9	2.0
M1124-15	2567	2757	3578	68	1.1	3.1	2.9	4.1	7.1	1.6	1.8
Commercial hybrids											
CH9	2568	2440	3102	69	0.7	3.2	3.4	4.3	7.6	2.6	2.2
CH4	2321	3273	4588	72	0.8	3.2	3.2	4.2	6.9	2.0	2.4
CH8	2297	1398	2719	71	0.8	3.4	3.3	4.0	8.0	1.7	1.9
CH3	2032	1952	2403	72	0.6	3.7	3.2	3.7	6.7	2.2	2.2
CH1	1767	1702	2724	69	0.7	3.7	3.1	4.4	6.5	1.9	1.9
CH6	1711	2716	3389	69	0.8	3.5	2.9	3.8	7.5	1.9	2.0
CH7	1234	2185	3566	67	0.7	3.9	2.9	4.0	7.7	2.3	2.2
CH5	1072	3207	4610	69	0.7	3.8	3.5	4.4	6.5	2.6	2.4
CH2	1016	1199	2461	69	0.6	4.1	3.4	4.3	7.4	1.9	2.3
CH10	1462	1746	3301	69	0.8	3.8	3.3	4.5	7.7	2.2	2.1
Local check	1241	939	1276	71	0.7	3.7	3.8	4.1	7.3	2.3	2.3
Mean	2174.4	19.4	3822.8	68.7	0.8	3.4	3.0	4.0	7.3	2.0	2.0
LSD (0.05)	1190.2	975.9	1101.27	3.2	0.2	0.6	0.6	0.5	1.0	0.6	0.4
CV (%)	41.7	2414.1	19.11	4.1	20.85	14.72	13.2	11.9	11.7	18.8	16.5
Prob<	***	***	***	***	***	***	***	***	**	***	**
Hybrid x Year	**	**	ns	*	**	*	***	ns	**	***	*

*, **, ***Significant at 0.5, 0.01 and 0.001 probability levels, respectively;

a Ear aspect, scored on a scale of 1–5, where 1 = uniform ear filled and grain color and 5 = poor ear filling with multiple grain colors;

b Plant aspect, a visual score on a scale of 1–5, where 1 = good phenotypic appeal and 5 = poor phenotypic appeal;

c1 First leaf senescence scored on a scale of 1–10, where 1 = 10% leaf death and 10 = 100% leaf death;

c2 Second leaf senescence scored on a scale of 1–10, where 1 = 10% leaf death and 10 = 100% leaf death;

d Tassel blast scored on a scale on a scale of 1–5, where 1 = few tassel blast and 5 = dry tassel;

e Barren plants scored on a scale of 1–5, where 1 = few barren plants and 5 = all plants without ears; DSHTS, combined drought and heat stress; DS, drought stress; WW, well-watered.

Our results showed that five of the best 10 DT hybrids produced 9% to 26% more grain yields than the best commercial hybrid (CH9) and had yield advantage of 56% to 64% over the local check under DSHTS (Table 7). The best 10 DT hybrids were comparable to four commercial hybrids (CH4, CH6, CH8, and CH9) under DS and were also competitive with five commercial hybrids (CH4, CH5, CH6, CH7, and CH9) under WW conditions. The best DT hybrids had higher EPP and fewer TB than the commercial hybrids and the local check. Most of them also had good plant type and ear aspect, as well as few BP under DSHTS condition and were similar to the commercial hybrids in days to silking, the first and second leaf death scores.

## 4. Discussion

When considering the importance of identifying maize genotypes with high levels of tolerance to combined drought and heat stress for climate change adaptation, we screened promising drought tolerant hybrids under DS and DSHTS. The significant differences found among DT hybrids for grain yield and several other agronomic traits recorded under both DS and DSHTS, indicate the presence of genotypic difference among promising DT hybrids. The significant interactions of hybrids with environments for grain yield and other agronomic traits suggest the influence of DS and DSHTS on the expression of these traits. The greatest influence of both DS and DSHTS occurred in mid-April each year, during the three weeks around flowering and grain-filling stages. These two stages are the most critical period determining yield potential in maize.

The extent of yield loss is dependent on severity of drought and heat stress, field environment and the maize genotypes under study. In this study, drought stress reduced grain yield by 58%, while DSHTS reduced grain yield by 77%, agreeing with previous findings that demonstrated more yield loss from combined effects of drought and heat stress than drought stress alone [6,22,23]. Combined drought and heat stress adversely affected grain yield and days to flowering of most hybrids more than drought, possibly due to its adverse effects on pollen production, or ovule fertility, leading to premature embryo abortion and reduced grain weight [32]. Our results corroborate earlier findings [20,32,33] that the effect of DSHTS was higher in maize at the reproductive stage than at the vegetative stage.

Previous studies in maize [4,34] and wheat (*Triticum aestivum* L.) [35] showed that tolerance to DSHTS is genetically distinct from tolerance to individual drought and heat stresses. However, our results indicated that breeding for DS, to some extent, has spill-over effect under DSHTS, with 50% of the best DT hybrids selected under DS showing appreciable grain yields under DSHTS. Some DT hybrids that produced high grain yields under both DS and DSHTS were found in the present study, implying that in a short term, breeders should screen available DT varieties under DSHTS to identify those with high levels of tolerance to combined drought and heat stress quickly for further testing and release, as well as for use as donors of DSHTS.

The observed significant correlations between the same traits measured under DS, DSHTS as well as WW conditions suggest the presence of common genetic factors controlling expression of these traits under the three growing conditions. However, the observed weak correlations between grain yield under DS and DSHTS indicate the presence of independent genetic factors controlling yield performance under these conditions, which agrees with previous studies [4,34]. The significant association of grain yield under DS with yield under WW observed in our study is consistent with the findings of other researchers [4,36]. This result, however, contradicts the findings of Trachsel et al. [34] who reported very weak correlation (*r* = 0.075) between grain yield under optimum and that under stressed conditions.

The strong correlation between grain yield and TB and BP demonstrated that these traits together with other secondary traits (ASI, EPP, ear and plant aspect cores, days to anthesis, and silking) should be included in an index to select promising genotypes under DSHTS. Secondary traits that are strongly correlated with grain yield suggest that they function together in determining the plants’ response to combined stress [37]. Although we found weak (0.13) repeatability for TB relative to grain yield under DSHTS in our study, this trait has the potential to improve the selection efficiency for grain yield under DSHTS. Other researchers [38] also found weak heritability for ASI relative to grain yield, but suggested its use for selection under DS [39–42].

Knowledge of the relationships among DT hybrids developed from diverse genetic backgrounds can help to identify a set of specific DT hybrids that have good levels of tolerance to DS and DSHTS. Grain yield and morphological (adaptive secondary) traits were used to estimate these relationships. Under DSHTS, group G-2 clustered DT hybrids tolerant to DSHTS, including two commercial hybrids (CH6 and CH9), which were competitive with the best DT hybrid, suggesting that these hybrids had common adaptive secondary traits that were better expressed under DSHTS as predicted by dendrogram. The majority of DT hybrids and commercial hybrids grouped in G-1 showed high levels of susceptibility to DSHTS with low grain yields. However, seven of the susceptible hybrids (M1124-27, M1227-5, M1227-6, M1227-7, M1227-9, M0826-1, CH5), including a released hybrid and commercial hybrid were grouped (G-1) with best DT hybrids under DS, suggesting that some DT hybrids that are susceptible to DSHTS perform better under DS condition.

Although we observed few DT hybrids with desirable traits grouped together in the dendrogram, most desirable agronomic traits were not found in a single DT hybrid, suggesting the need for multiple traits pyramiding by introgressing the desired traits into the genetic background of adapted DT hybrids. Secondary traits, like EPP, TB, ear and plant aspect scores, days to anthesis, and silking that were found to be strongly correlated with grain yield under stressed conditions can be combined with other traits, such as canopy temperature and leaf firing for field screening. Alam et al. [43] suggested that traits that are indicative of reproductive success under HTS, including ASI, TB, tassel sterility, pollen viability, and stigma receptivity and other morpho-physiological traits, including leaf firing, senescence, and chlorophyll content need to be used along with grain yield for selecting stress tolerant germplasm. Most of these traits are simple and rapid to measure in large number of progenies and are used for selecting for tolerance to DSHTS.

We observed that some productive DT hybrids were tolerant to DSHTS as well as under WW conditions. However, some DT hybrids together with a commercial hybrid CH7 ranked among the worst yielders under DSHTS, with grain yields well below the trial mean, suggesting that not all DT hybrids are tolerant to DSHTS as found in other studies [4,34,35]. We identified three DT hybrids, M1227-17, M0826-3, and M1124-18, among the top 10 hybrids with consistently high grain yields under both DS and DSHTS. These hybrids combined high grain yields with least TB and BP and increased EPP under stress conditions. M0826-3 is a released DT hybrid in Nigeria that also has attributes of resistance to maize streak virus and tolerance to low soil nitrogen [44].

Although we have not designed an experiment specifically for heat stress, the level of tolerance observed in some DT hybrids under DSHTS may be attributed to the effects of the relatively high day temperatures (32–37 ^°^C) when the hybrids used in this study were selected under DS at Ikenne during the dry season. This agrees with Sehgal et al. [37] who observed combined tolerance to drought and heat stress in lentil (*Lens culinaris* Medic.) genotypes evaluated under DS, HTS, and DSHTS. Limited information is available on performance of DT maize hybrids under DSHTS, which need further investigation to understand the relationship between tolerance to DS and DSHTS and the genetic and biological mechanisms that control tolerance to DSHTS in maize. Comprehensive study by the use of a combination of conventional and molecular breeding tools is needed to understand the mode of inheritance of adaptive secondary traits that confer tolerance to DSHTS to enhance breeding scheme for DSHTS. Genotypes showing tolerance to DSHTS will be used as a source for the development of new inbred lines. In addition, stress tolerant maize germplasm introduced from diverse sources should be screened to identify source materials for developing new multiple-stress tolerant inbred lines with higher levels of tolerance to DSHTS.

## 5. Conclusions

In conclusion, our study revealed that the combined effects of drought and heat stress at flowering and grain filling stages were detrimental to maize grain yield. Large variations were observed in grain yield and other agronomic traits among the DT hybrids under DSHTS condition. The effects of DSHTS on grain yield were more on commercial hybrids than on DT hybrids. The weak correlation of grain yield under DSHTS with that under DS and WW suggest the complexity of genetic control of yield under DSHTS in maize. The strong correlations between grain yield and some secondary traits, including ear and plant aspect scores and EPP under both DS and DSHTS, suggest the existence of common genetic factors controlling these traits that may be used to enhance productivity under the two stresses. Evaluation of promising DT hybrids under DSHTS, DS, and WW allowed for the identification of three high yielding DT hybrids (M1227-17, M0826-3, and M1124-18) with high levels of tolerance to DSHTS. The parents of these hybrids can be used as source materials for developing inbred lines with higher levels of tolerance to DSHTS. The top five hybrids under DSHTS with yield advantages of 9% to 25% over the best commercial hybrid are suitable for production in tropical lowlands where DSHTS is of common occurrence in farmers’ fields. The study highlighted the need to modify the screening techniques and the addition of more adaptive traits, including leaf canopy temperature and application of high throughput phenotyping and genotyping techniques to enhance the understanding of DSHTS mechanisms and their genetic basis of inheritance to improve the breeding scheme for tolerance to combined drought and heat stress.
